# Self-disgust in patients with borderline personality disorder. The associations with alexithymia, emotion dysregulation, and comorbid psychopathology

**DOI:** 10.1186/s40479-023-00232-1

**Published:** 2023-08-29

**Authors:** Emilia Kot, Barbara Kostecka, Joanna Radoszewska, Katarzyna Kucharska

**Affiliations:** 1 Department of Neuroses, Personality Disorders, and Eating Disorders, Institute of Psychiatry and Neurology, 9 Sobieskiego Street, Warsaw, 02-957 Poland; 2 II Department of Psychiatry, Medical University of Warsaw, 8 Kondratowicza Street, Warsaw, 03-242 Poland; 3 Faculty of Psychology, University of Warsaw, 5/7 Stawki Street, Warsaw, 00-183 Poland; 4 Institute of Psychology, Cardinal Stefan Wyszyński University in Warsaw, 1/3 Wóycickiego Street, Warsaw, 01-938 Poland

**Keywords:** Personality disorders, Disgust, Emotion regulation, Depression, Anxiety, Self-conscious emotions

## Abstract

**Background:**

Self-disgust is a negative self-conscious emotion, which has been linked with borderline personality disorder (BPD). However, it has not yet been investigated in relation to both emotion dysregulation and alexithymia, which are recognized as crucial to BPD. Therefore, the aim of our study was to measure these variables and examine the possible mediational role of emotional alterations and comorbid anxiety and depression symptoms in shaping self-disgust in patients with BPD and healthy controls (HCs).

**Methods:**

In total, the study included 100 inpatients with BPD and 104 HCs. Participants completed: the Self-Disgust Scale (SDS), Disgust Scale – Revised (DS-R), Toronto Alexithymia Scale (TAS-20), Emotion Dysregulation Scale short version (EDS-short), Borderline Personality Disorder Checklist (BPD Checklist), State-Trait Anxiety Inventory (STAI), and Center for Epidemiologic Studies Depression Scale (CESD-R).

**Results:**

Inpatients with BPD showed higher self-disgust, alexithymia, emotion dysregulation, core and comorbid symptoms levels, and lower disgust sensitivity. Alexithymia, emotion dysregulation, and trait anxiety partially mediated between BPD diagnosis and self-disgust. The relationship between the severity of BPD symptoms and self-disgust was fully mediated by alexithymia, emotion dysregulation, depressive symptoms, and trait anxiety.

**Conclusions:**

The results of our study may imply the contribution of emotion dysregulation, alexithymia, and comorbid psychopathology to self-referenced disgust in BPD.

## Background

Borderline personality disorder (BPD) is characterized by impairments in self-functioning, including distorted identity, a persistent pattern of unstable interpersonal relationships, excessive efforts to avoid abandonment, enduring feeling of emptiness, affective instability, behavioral disinhibition, as well as recurrent threats or episodes of self-harm [1]. BPD is distinguished by the presence of affective instability in addition to negative emotionality, which sets it apart from many other mental disorders [2]. It is presumed that emotion dysregulation may lie at the core of the disorder [3, 4]. A disturbed self-image is also a common BPD feature, often manifested by self-criticism, self-hate, and a feeling of disgust towards aspects of the self [5, 6].

### Abnormalities within experiencing disgust in BPD

Several lines of research suggest that individuals with BPD may be afflicted by alterations within experiencing disgust, which may include increased disgust sensitivity (i.e., the intensity of experiencing disgust) [7, 8], elevated disgust propensity (i.e., one’s general tendency to experience disgust) [9], and a raised level of self-directed disgust [10]. Impairment in recognizing negative emotional expressions, including the expression of disgust [11], has been observed in individuals with BPD. However, some studies have shown enhanced facial disgust recognition [9, 12]. Ecological momentary assessment studies revealed that individuals with BPD experienced state disgust more frequently, but the results regarding its intensity are diverse, indicating either increased or unchanged levels [13, 14]. Interestingly, patients with BPD did not differ in the frequency and intensity of disgust experience from patients with bulimia and patients with posttraumatic stress disorder, which suggests transdiagnostic patterns of disgust [13]. In BPD, disgust abnormalities may be related to anxiety and paranoid symptoms, both of which are associated with an elevated disgust reactivity [8]. Abnormalities within disgust have also been linked to altered emotional regulation and interpersonal relationships due to the significance of disgust in interpersonal context [15]. Moreover, it is possible that emotion dysregulation in BPD, especially featured in terms of negative emotions such as anxiety, anger, sadness, or shame [14], may extend to disgust [7, 16] and to disgust-related regulation deficit [9].

### Self-disgust in BPD

BPD is specifically characterized by a fragile self-representation, an impoverished and/or unstable self-structure, self-loathing, and challenges in differentiating between self and others [17]. Therefore, it has been suggested that self-related disgust may be crucial for BPD and its treatment [7]. Self-disgust is a self-conscious emotion schema characterized by loathing and revulsion at the experienced self, body, and one’s actions, with the particular meaning of behaviors that are inconsistent with the desired self [18]. It might be considered a result of the interplay between one’s predisposition to experience disgust, internalization of socially comparative processes, and changes in self-concept over time [19]. Self-disgust tends to remain relatively constant and is not easily changeable [20]. Individuals with mental disorders exhibit elevated levels of self-disgust [21], and this is predicted by the severity of psychological problems. Moreover, it has been found that patients with eating disorders and those with BPD may have higher levels of self-disgust compared to patients with other mental disorders [10].

The extant research confirms the increased level of self-disgust in BPD [9, 10, 22] and its positive association with general BPD symptom severity [9, 23] as well as with the level of dysfunctional behavior [23]. Klonsky & Muehlenkamp [24] proposed that self-injury behavior (nonsuicidal self-injury; NSSI), which occurs in the majority of patients with BPD, may serve to express self-loathing. Consistently, a positive association between self-disgust and NSSI was observed in patients with BPD [23]. In a general sample, self-disgust mediated between depressive symptoms/sexual abuse and NSSI [25]. It was also identified as a relevant predictor of suicide risk [26]. In a study using neuroimaging methods, it was found that NSSI and self-disgust were negatively associated with the volume of the secondary somatosensory cortex (i.e., a brain area engaged in the processing of pain, tactile and visceral sensations) in patients with BPD, which could be a result of deficits in the integration of sensory inputs [27]. Additionally, in patients with BPD, self-disgust was found to be positively associated with the volume of the insula, a structure that is of crucial importance for disgust processing [27] and self-awareness [28], which, given the role of identity disturbance in BPD, could lead one to hypothesize a possible neural basis of alterations in self-disgust [23].

Although there is a connection between BPD symptoms and self-disgust, the precise role and whether self-disgust is primary or secondary to BPD remain unclear. According to Linehan, BPD is a result of the interaction between biological factors and environmental influences (i.e., emotional invalidation or emotional abuse) [6]. These factors may result in subsequent development of poor emotion recognition (i.e., alexithymia) and emotion dysregulation [29], but they may also constitute risk factors for the development of self-disgust [21]. In line with this perspective, longitudinal studies have shown that affective instability is a strong predictor of future BPD characteristics in a general sample [30]. Crowell et al. [6], proposed that the sustained biology-environment interaction leads to the intensification of extreme emotional displays and reinforcement of emotional lability, increasing the risk of emotion dysregulation. Emotion dysregulation becomes trait-like and leads to the conditioning of negative emotional (e.g., shame), cognitive (e.g., self-hatred), behavioral (e.g., impulsive behaviors), and social (e.g., social isolation) outcomes. Furthermore, these negative outcomes become emotion regulation or avoidance strategies, which, in turn, exacerbate the underlying negative individual-environment transaction. In this model, self-disgust, an enduring self-conscious construct developed through repeated negative experiences and self-evaluations, may serve as a developmental factor for BPD-related symptoms and also emerge as a negative outcome due to emotional dysregulation in individuals with elevated BPD symptoms. In line with this idea, Krawitz [31] proposed that self-loathing in BPD may arise as a conditioned response within a vicious cycle, reinforced by identity and emotional alterations.

Regarding studies exploring the causal aspect of the associations between self-disgust and psychopathological symptoms in BPD, their results are inconclusive. Most of the studies posit self-disgust as an antecedent of BPD-related problems [32] or as a mediator between cognitive, emotional, or behavioral constructs and BPD [33, 34]. On the other hand, Ille et al. [10] based on their results suggested that disorder-associated symptoms pose a particular risk for elevated self-disgust in patients with BPD. In line with this, Lazuras et al. [35] showed that non-planning impulsivity had significant indirect effects on self-disgust via emotion regulation strategies and self-regulation in a general sample, suggesting that when greater impulsivity is displayed and self-regulation fails, there is an increased likelihood of experiencing heightened self-disgust.

#### Assessing the link between emotion dysregulation and alexithymia with self-disgust

Emotion dysregulation in BPD encompasses emotion sensitivity, heightened and labile negative affect, deficit of appropriate regulation strategies, and surplus of maladaptive regulation strategies [3]. As mentioned earlier, emotion dysregulation may contribute to the development and maintenance of core BPD symptoms and other negative cognitive, emotional, and behavioral outcomes related to BPD [4, 6]. Research has shown that in patients with BPD, emotion dysregulation and trait anger sequentially mediate the association between the severity of BPD symptoms and aggression [36]. Moreover, individuals with BPD may engage in more frequent attempts to regulate emotions, however, these efforts are not accompanied by a corresponding increase in perceived effectiveness [37]. This finding may lead one to assume that it could contribute to a predominantly negative self-concept in BPD and perhaps also to self-disgust.

Alexithymia is a subclinical trait characterized by a cognitive deficit in experiencing emotions, specifically difficulty identifying and expressing one’s own emotions, difficulty distinguishing between emotions and physical sensations, and an externally oriented thinking style [38]. It seems to be associated with altered interoceptive awareness and a tendency to misinterpret bodily signals [39]. Importantly, alexithymia is associated with low mentalizing and emotion dysregulation. It is a clinically prominent phenomenon in BPD [40], and since the ability to recognize and mentalize emotions is a prerequisite for their effective regulation, the findings on the link between alexithymia and deficits in emotion regulation are not surprising [41, 42]. Despite the scarcity of direct evidence linking alexithymia to self-disgust in BPD, it could be hypothesized that they might be associated given the conceptualization of self-disgust as a result of difficulty in distinguishing between self and non-self and recognizing parts of the self that may lead to self-rejection [18].

## Methods

### Aims and hypotheses

The results of previous studies consistently indicate an increased level of self-disgust in individuals with BPD [21] and its association with the increased severity of BPD symptoms [9]. However, most of the extant studies had limited patient sample sizes [10, 23], and the majority of them considered self-disgust as a factor preceding emotional problems in BPD [32] or as a construct reinforcing the influence of cognitive, emotional, or behavioral difficulties on BPD features [34]. Nevertheless, it has been mentioned that disgust towards self might be particularly prominent in BPD [10], and it might possibly be exacerbated by the symptoms characteristic of BPD, such as impulsivity, difficulties with self-regulation, and maladaptive emotion regulation strategies [35]. Given these points, the relative stability of self-disgust over time [43], and the understanding of self-disgust as a negative outcome that may occur as a result of adverse experiences, self-evaluations, and emotional alterations, we aimed to examine self-disgust in relation to alexithymia and emotion dysregulation and its connection to BPD.

The specific aims of our study were as follows: (1) to compare self-disgust between a large clinical sample of patients with BPD and HCs, with the hypothesis that self-disgust will be increased in the patient group; (2) to compare levels of disgust sensitivity, BPD symptoms, emotion dysregulation, and alexithymia, as well as comorbid depressive and anxiety symptoms between patients with BPD and HCs, with the expectation that the level of the above variables would be higher in the patient group; and (3) to assess the associations between self-disgust and the above-mentioned constructs in both groups. Previous studies have shown moderate-to-large associations between self-disgust and depression and anxiety [44]. Additionally, considering that disgust sensitivity has been associated with both self-disgust and BPD psychopathology [9, 18, 23], and that disgust or contamination-based appraisals are deemed necessary to generate self-disgust [21], we decided to include this construct in our study. We hypothesized that self-disgust will be positively associated with emotional dysfunctions, core and comorbid psychopathology, and disgust sensitivity in both examined groups.

As mentioned earlier, existing research suggests that disgust alterations in BPD might be associated with anxiety [8], emotion dysregulation [7, 16], the severity of BPD symptoms, and the level of dysfunctional behavior [23]. Moreover, path analyses have shown that BPD, depression, and anxiety symptoms may mutually reinforce one another concurrently and over time [45]. Additionally, some research indicates a relationship of alexithymia with emotion dysregulation and BPD symptoms [29]. Therefore, considering the revealed associations between the constructs examined in our study and the knowledge on the possible correlates of increased self-disgust in BPD, we aimed to extend the extant research by (4) exploring the mediational role of emotional dysfunctions as well as comorbid psychopathology in the relationship between BPD diagnosis or BPD symptom severity and the level of self-disgust. We expected that emotion dysregulation, alexithymia, and comorbid symptoms will indirectly account for the association between BPD diagnosis/BPD symptom severity and self-disgust.

### Participants

Our initial sample included 213 participants; however, 7 participants were excluded from the HCs group due to increased levels of psychopathological symptoms. One participant from the HCs group and 1 from the group of patients with BPD were excluded after outlier diagnostics. Thus, the final sample consisted of 204 participants – 100 women with BPD hospitalized at a specialized inpatient therapeutic ward at the Institute of Psychiatry and Neurology in Warsaw, Poland, and 104 women in the HCs group (including the students from the University of Warsaw, Poland) who were recruited via project invitations. All participants were of Polish origin and HCs were matched for age to the BPD group. Most patients were already diagnosed with BPD based on clinical interviews. The diagnosis was verified by clinical psychologists based on a structured clinical interview for personality disorders (SCID-II PD) [46]. It was subsequently consulted with and confirmed by a senior psychiatrist. Comorbid psychiatric diagnoses were allowed, except for antisocial personality disorder, schizophrenia-spectrum or other psychotic disorder, current mania or hypomania episode. Exclusion criteria for both groups encompassed: serious somatic diseases, neurodevelopmental disorders, intellectual disability, previous brain trauma, severe neurological dysfunction or diseases, and alcohol or substance dependence within the last 6 months. Subjects from the HCs group were informed about previous or current mental disorders as exclusion criteria.

Less than half of the BPD patients were diagnosed with eating disorders (*N* = 44). Depressive disorders were confirmed in 48 patients. Thirty-seven patients were diagnosed with anxiety or fear-related disorders and 10 patients were confirmed with obsessive-compulsive disorder. In general, patients aged between 18 and 45 years (*M* = 24.19; *SD* = 5.41) and HCs aged between 19 and 34 years (*M* = 24.94; *SD* = 3.70). No group differences were found for age, *F*(1, 202) = 1.35, *p* = 0.25.

### Measures

#### Clinical measures

The Borderline Personality Disorder Checklist (BPD Checklist [47]; Polish adaptation by Grzegorzewski [48]) is a 47-item self-report questionnaire to examine the subjective burden caused by BPD symptoms during the last month. It is based on the DSM-IV and DSM-5 criteria. The scores equal to or greater than 100 are consistent with a diagnosis of BPD. The scale consists of nine subscales: *Abandonment*, *Relationships*, *Identity*/*Self-concept*, *Impulsivity*, *Self-mutilation/Parasuicide*, *Mood*, *Emptiness*, *Anger*, *Paranoid/Dissociation*. The English version of the tool has very high internal consistency both for the group of patients with BPD (α = 0.92) and for the joint group of patients with various personality disorders and HCs (α = 0.97; [47]). In the current study, reliability estimates for BPD Checklist were α = 0.93 in BPD patients and α = 0.89 in HCs.

The State-Trait Anxiety Inventory (STAI; [49]; Polish adaptation by Wrześniewski et al. [50]) is a self-rated measure including 20 items assessing the current state of anxiety (STAI-State) and 20 items measuring a general disposition to be anxious (STAI-Trait). Answers are marked on a 4-point Likert scale, with higher scores indicating higher levels of anxiety. The Polish version of the tool has good internal consistency (STAI‐ State: α = 0.83–0.92 and STAI‐Trait: α = 0.86–0.92; [50]). In our samples, alpha coefficients in BPD patients for STAI-State was α = 0.93 and for STAI-trait it was α = 0.91. In HCs for STAI-state α = 0.89 and for STAI-Trait: α = 0.87).

The Center for Epidemiologic Studies Depression Scale–Revised (CESD-R; [51]; Polish adaptation by Koziara [52]) was used to examine depressive symptoms. The CESD-R is a 20-item self-report measure in which each item is rated on a 5-point Likert scale. Total scores range between 0 and 80, with higher scores indicating higher level of depressive symptoms. The Polish version of the tool has very good internal consistency (α = 0.95; [52]). In the current study, alpha coefficients for the CESD-R were α = 0.89 in BPD patients and α = 0.84 in HCs.

#### Emotional functioning

Emotional Dysregulation Scale, short version (EDS-short; [53]; Polish adaptation by Grzegorzewski, [54]) is a 12-item self-report questionnaire in which items are rated on a 7-point Likert scale. The items assess domains of *emotional experiencing*, *cognition*, and *behavior*. Higher scores are indicative for greater emotion dysregulation. The English version of the tool has very high internal consistency (α = 0.93–0.95). In the current study, the reliability estimates for the EDS-short scale were α = 0.90 for BPD patients and α = 0.89 for HCs.

Toronto Alexithymia Scale-20 (TAS-20, [55]; Polish adaptation by Ścigała et al. [56]) is a 20‐item self‐report questionnaire to measure the alexithymia construct in which items are rated on a 5-point Likert scale. It consists of three subscales – *difficulty describing feelings, difficulty identifying feeling*, and *externally-oriented thinking*. A total score out of 100 may be calculated. The greater the final score, the higher the level of alexithymia. A score equal to or greater than 61 may suggest that a person is alexithymic. The Polish version of the tool has good psychometric properties (α = 0.82–0.86). In our study, alpha coefficients for the TAS-20 were α = 0.75 in BPD patients and α = 0.80 in HCs.

#### Disgust experience

The Disgust Scale – Revised (DS-R; [57, 58]; Polish adaptation by Kot [59]) is a self-report scale including 25 items and 2 additional catch questions to identify poor responders. The DS-R assesses individual disgust sensitivity to diverse categories of objects and situations. The items are rated on 5-point scales both for the items 1–14 (0 – *strongly disagree*, 4 – *strongly agree*) and for the items 15–27 (0 – *not disgusting at all*, 4 – *extremely disgusting*). A total score out of 100 may be calculated. The DS-R consists of three subscales: Core Disgust (food, animals, body products), Animal-Reminder Disgust (death and body envelope violations), and Contamination Disgust (contagion transmission). The Polish version of DS-R demonstrated good internal consistency in a study involving patients with anorexia nervosa and HCs (for patients: α = 0.87; for HCs: α = 0.80; [59]). In the current study, the reliability estimates for the DS-R were α = 0.87 in BPD patients and α = 0.85 in the HCs group.

The Self-Disgust Scale (SDS; [60]; Polish adaptation by Kot [61]) is a self-report 12-item scale with additional six neutral filler items. The SDS measures the level of disgust directed toward the self and consists of three subscales: Appearance, General Self-Concept, and Behavior. Each item is rated on a 7-point Likert scale (1 – *strongly agree*, 7 – *strongly disagree*). A total score ranges from 12 to 84. The higher score, the greater self-disgust. The Polish version of SDS demonstrated good psychometric properties in a study involving patients with anorexia nervosa and HCs (for patients: α = 0.79; for HCs: α = 0.88; [61]). In the current study, alpha coefficients were α = 0.87 in BPD patients and α = 0.80 in HCs.

### Procedure

Study approval was obtained from the Bioethics Committee at the Institute of Psychiatry and Neurology in Warsaw, Poland (no. 26/2017). Subjects read the study description and signed the informed consent sheet prepared in concordance with the current version of the Declaration of Helsinki. Afterward, they filled in a battery of psychological measures described below.

### Statistical analyses

Statistical analyses were carried out using IBM SPSS Statistics 28 software. Before conducting the main analyses, we assessed the data for the presence of outliers. We also assessed the normality of distribution by inspecting histograms and Q-Q plots as well as the following criteria: absolute values for skewness and kurtosis lower than 2.00 [62]. If the data were non-normally distributed but met the above criteria for skewness and kurtosis, we analyzed them with a parametric test.

ANOVA and the Kruskal-Wallis test were performed to assess the differences between the compared groups in all of the analyzed variables. We have provided eta squared coefficients (*η*^*2*^) as an estimate of the effect size and used the metric of small: *η*^*2*^ = 0.01–0.05, medium *η*^*2*^ = 0.06–0.13, and large *η*^*2*^ ≥ 0.14 effect size.

Because the absolute values for skewness and kurtosis for most of the examined variables were lower than 2.00, Pearson’s correlations were performed. For the variables that did not meet the abovementioned criteria, it was ascertained that the value of Spearman’s rho coefficient reached similar values as Pearson’s correlation coefficient [63]. Correlations between self-disgust, disgust sensitivity, BPD symptoms, emotion dysregulation, alexithymia, state and trait anxiety, and depression scores were computed. We employed the metric of small: *r* = 0.10–0.29, medium: *r* = 0.30–0.49, and large: *r* ≥ 0.50 effect sizes. Correlation analyses were performed separately for each group.

Further, multiple regression analyses were conducted. An analysis of standard residuals was carried out, which showed that the data from the BPD and HCs groups contained two outliers. After removing the outliers the data met the required assumptions (BPD: standard residual min = − 2.62, standard residual max = 1.82; HCs: standard residual min = − 2.36, standard residual max = 2.79). The data also met the assumption of independent errors (Durbin–Watson value: BPD: 1.91; HCs = 1.95). The histogram of standardized residuals indicated that the data contained approx. normally distributed errors, as did the normal P–P plots of standardized residuals. The scatterplots of standardized predicted values showed that the data met the assumptions of homogeneity of variance. The data were also checked for multicollinearity (BPD: VIF = 1.22–2.14, Tolerance = 0.47–0.82; HCs: VIF = 1.42–2.48, Tolerance = 0.40–0.70). Some variables were non-normally distributed in both groups. However, the absolute values for skewness and kurtosis were below 2.00, therefore, all values were deemed acceptable and they were entered into the regression models without log-transformation.

Subsequently, parallel mediation analyses were conducted to test the mediating effect of emotion dysregulation, alexithymia, depressive symptoms, and trait anxiety on the relationship between BPD diagnosis (HCs group vs. BPD group) or BPD symptoms severity and self-disgust. We performed mediation analysis procedures with bootstrap sampling, as recommended by Preacher and Hayes [64]. The bootstrap method estimates indirect effects through one or more mediator variables with bias-corrected bootstrap confidence intervals. The analysis includes the estimation of direct, indirect, and total effects. In this study, the direct path was the effect of BPD diagnosis or BPD symptoms severity on self-disgust, independent of their effects on mediating variables. The indirect effects were the paths linking BPD diagnosis/BPD symptoms severity to self-disgust via emotion dysregulation, alexithymia, depressive symptoms, and trait anxiety. The total effects of BPD diagnosis/BPD symptoms severity on the level of self-disgust were the sums of the direct and indirect effects. The analyses were conducted using the PROCESS macro [65]. Five thousand bootstrap resamples were used to generate bias-corrected 95% confidence intervals for the indirect effect. As outlined by Preacher and Hayes [64], mediation is demonstrated when the indirect effect is statistically significant and the confidence intervals do not contain zero (i.e., indicating that the indirect effect is significantly different from zero). All tests were two-tailed and considered statistical significance at *p* < 0.05.

## Results

### Group differences in self-disgust, disgust sensitivity, BPD symptoms, emotion dysregulation, alexithymia, state and trait anxiety, and depressive symptoms

Patients with BPD reported higher levels of self-disgust and all of its components as compared to HCs (see Table 1). In turn, lower levels of overall disgust sensitivity, animal-reminder disgust, and contamination disgust were observed in women with BPD compared to HCs. Importantly, the effect size for disgust sensitivity indicators was small, while for self-disgust and its dimensions, it was large. Further, BPD patients showed higher levels of BPD symptoms, emotion dysregulation, alexithymia, state and trait anxiety, as well as depressive symptoms (with large effect sizes).

**Table 1:** Differences between BPD and HCs groups in self-disgust, disgust-sensitivity, BPD symptoms, emotion dysregulation, alexithymia, state and trait anxiety, and depressive symptoms, based on the results of ANOVA and the Kruskal-Wallis test

	BPD group (*N* = 101)		HCs group (*N* = 104)		*F* or *χ²*	95% CI	*η*^*2*^ or *ε*^*2*^
	*M*	*SD*	*M*	*SD*			
SDS total score	59.71	12.08	27.34	9.69	* F* = 447,43***	[29.36; 35.39]	*η*^*2*^ = 0.69
SDS General Self-Concept	20.84	4.58	9.85	3.85	* F* = 345.07***	[9.82; 12.16]	*η*^*2*^ = 0.63
SDS Appearance	19.88	4.71	9.66	4.08	* F* = 274.36***	[9.00; 11.43]	*η*^*2*^ = 0.58
SDS Behavior	18.99	4.93	7.83	3.66	* F* = 338.97***	[9.97; 12.37]	*η*^*2*^ = 0.63
DS-R total score	1.95	0.73	2.17	0.60	* F* = 5.50*	[-0.40; -0.03]	*η*^*2*^ = 0.03
DS-R Core Disgust	2.33	0.74	2.36	0.61	* F* = 0.10	[-0.22; 0.16]	*η*^*2*^ = 0.001
DS-R Animal Reminder Disgust	1.80	1.07	2.27	0.94	* F* = 11.19***	[-0.75; -0.19]	*η*^*2*^ = 0.05
DS-R Contamination-Based Disgust	1.29	0.79	1.56	0.74	* F* = 6.29*	[-0.48; -0.06]	*η*^*2*^ = 0.03
BPD Checklist total score	135.16	28.88	66.70	13.50	* F* = 476,14***	[62.27; 74.64]	*η*^*2*^ = 0.70
BPD Abandonment	22.74	6.15	9.77	2.86	* F* = 377.98***	[11.66; 14.29]	*η*^*2*^ = 0.65
BPD Relationships	8.75	3.33	4.25	1.49	* F* = 157.47***	[3.79; 5.21]	*η*^*2*^ = 0.44
BPD Identity/Self-concept	28.34	6.21	12.61	4.10	* F* = 459.75***	[14.29; 17.18]	*η*^*2*^ = 0.70
BPD Impulsivity	15.95	5.14	11.38	2.82	*χ²* = 58.60***	[3.44; 5.71]	*ε*^*2*^ = 0.29
BPD Self-mutilation/Parasuicide	6.48	3.16	3.05	0.26	*χ²* = 122.60***	[2.82; 4.05]	*ε*^*2*^ = 0.60
BPD Mood	16.08	3.38	7.13	2.39	* F* = 478.69***	[8.13; 9.75]	*η*^*2*^ = 0.70
BPD Emptiness	4.35	0.97	2.03	0.91	*χ²* = 122.61***	[2.06; 2.58]	*ε*^*2*^ = 0.60
BPD Anger	10.10	3.83	5.09	1.18	*χ²* = 102.63***	[4.24; 5.79]	*ε*^*2*^ = 0.51
BPD Paranoid/Dissociation	22.28	6.45	11.40	2.86	* F* = 245.54***	[9.49; 12.27]	*η*^*2*^ = 0.55
EDS	68.30	13.42	35.61	12.94	* F* = 313.70***	[29.05; 36.34]	*η*^*2*^ = 0.61
TAS-20	64.29	10.25	43.86	10.47	* F* = 198.22***	[17.57; 23.30]	*η*^*2*^ = 0.50
STAI-State	55.07	12.88	34.04	8.23	* F* = 197.24***	[18.08; 23.98]	*η*^*2*^ = 0.49
STAI-Trait	61.54	7.89	39.43	8.23	* F* = 382.84***	[19.88; 24.34]	*η*^*2*^ = 0.66
CESD-R	49.24	14.19	14.14	8.42	* F* = 466.10***	[31.90; 38.31]	*η*^*2*^ = 0.70

*Note.* 95% CI = 95% confidence interval for the mean difference across the compared groups for ANOVA or 95% confidence interval for the median difference across the compared groups for the Kruskal-Wallis test; SDS – Self-Disgust Scale; DS-R – Disgust Sensitivity Scale; BPD Checklist – Borderline Personality Disorder Checklist; EDS – Emotional Dysregulation Scale; TAS-20 – Toronto Alexithymia Scale; STAI-State – state anxiety measured by the State-Trait Anxiety Inventory; STAI-Trait – trait anxiety measured by the State-Trait Anxiety Inventory; CESD-R – The Center for Epidemiologic Studies Depression Scale–Revised.

*p < 0.05. **p < 0.01. ***p < 0.001

### Associations of disgust variables, emotion dysregulation, and clinical psychopathology across groups

Self-disgust and its dimensions were not statistically significantly associated with disgust sensitivity or its components in any of the groups (p > 0.05), apart from a positive weak correlation between self-concept domain of self-disgust and core disgust in patients with BPD and between behavioral self-disgust and animal-reminder disgust in HCs group.

The overall pattern of relationships between self-disgust and BPD symptoms was similar across the groups and the associations between self-disgust and the level of symptoms of identity disturbance, mood alterations, feeling of emptiness, and total score for BPD symptoms were medium to large in both groups. Also, positive associations were found between self-disgust and emotion dysregulation, state and trait anxiety, and depressive symptoms in both examined groups (see Table 2).

**Table 2:** Associations between self-disgust, disgust sensitivity, core and comorbid psychopathology measures in the BPD group and in the HCs group

	BPD group				HCs group			
	SDS	SDS Appearance	SDS Self-concept	SDS Behavior	SDS	SDS Appearance	SDS Self-concept	SDS Behavior
BPD Checklist total score	0.41***	0.28**	0.34***	0.43***	0.55***	0.42***	0.48***	0.47***
BPD Abandonment	0.26**	0.14	0.20*	0.33***	0.41***	0.35***	0.30**	0.38***
BPD Relationships	0.38***	0.22*	0.28**	0.45***	0.13	0.12	0.10	0.10
BPD Identity/Self-concept	0.45***	0.24*	0.42***	0.48***	0.58***	0.43***	0.53***	0.50***
BPD Impulsivity	0.15	0.16	0.11	0.13	0.22*	0.14	0.19	0.23*
BPD Self-mutilation/Parasuicide	0.34***	0.34***	0.22*	0.29**	0.17	0.06	0.18	0.19
BPD Mood	0.32***	0.28**	0.25*	0.29**	0.39***	0.26**	0.41***	0.32***
BPD Emptiness	0.46***	0.37***	0.46***	0.34***	0.45***	0.47***	0.33***	0.30**
BPD Anger	0.27**	0.17	0.27**	0.26**	0.33***	0.23*	0.34***	0.27**
BPD Paranoid/Dissociation	0.28**	0.18	0.21*	0.32***	0.43***	0.34***	0.37***	0.37***
EDS	0.48***	0.35***	0.44***	0.43***	0.52***	0.38***	0.50***	0.42***
TAS-20	0.35***	0.33***	0.30**	0.27**	0.46***	0.38***	0.39***	0.39***
STAI-State	0.27**	0.28**	0.22*	0.19	0.42***	0.40***	0.41***	0.24*
STAI-Trait	0.50***	0.42***	0.45***	0.41***	0.65***	0.55***	0.56***	0.52***
CESD-R	0.41***	0.40***	0.29**	0.36***	0.46***	0.37***	0.45***	0.32***
DS-R	0.04	0.01	0.13	–0.03	0.07	0.10	0.15	0.11
DS-R Core	0.13	0.08	0.21*	0.05	0.07	0.09	0.06	0.02
DS-R Animal reminder	–0.02	–0.04	0.06	–0.06	0.18	0.06	0.18	0.20*
DS-R Contamination	–0.08	–0.04	–0.02	–0.13	0.08	0.07	0.13	–0.01

*Note.* SDS – Self-Disgust Scale; BPD Checklist – Borderline Personality Disorder Checklist; EDS – Emotional Dysregulation Scale; TAS-20 – Toronto Alexithymia Scale; TAS-20 DDF – difficulty describing feelings subscale; TAS-20 DIF – difficulty identifying feeling subscale; TAS-20 EOT – externally-oriented thinking subscale; STAI-State – state anxiety measured by the State-Trait Anxiety Inventory; STAI-Trait – trait anxiety measured by the State-Trait Anxiety Inventory; CESD-R – The Center for Epidemiologic Studies Depression Scale–Revised; DS-R – Disgust Sensitivity Scale.

* *p* < 0.05. ** *p* < 0.01. *** *p* < 0.001

### Prediction of self-disgust across groups

Results of the multiple regression analyses indicate that in the BPD group together trait anxiety, depressive symptoms, emotion dysregulation, alexithymia, and BPD symptoms, accounted for a significant proportion of the variance in self-disgust level, *F*(4, 95) = 13.34, *p* < 0.001, *R*^2^ = 0.36, adjusted *R*^2^ = 0.33. Depressive symptoms, emotion dysregulation, and alexithymia were significant predictors of self-disgust. However, within the same model in HCs, only trait anxiety significantly predicted the level of self-disgust, *F*(4, 99) = 22.11, *p* < 0.001, *R*^2^ = 0.47, adjusted *R*^2^ = 0.45 (see Table 3).

**Table 3:** Multiple linear regressions analysis predicting self-disgust as a function of trait anxiety, depressive symptoms level, emotion dysregulation, alexithymia, and BPD symptoms in the BPD group and in the HCs group

BPD group (n = 100)							
	B	*SE*	*Β*	*t*	95% CI for B	Adjusted R²	*F*
Constant	4.01	9.10		0.44	[–14.06, 22.07]	0.33***	10.61***
STAI-Trait	0.31	0.18	0.20	1.67	[–0.06, 0.68]		
CESD-R	0.19	0.09	0.22	2.10*	[0.01, 0.36]		
EDS	0.23	0.10	0.26	2.33*	[0.03, 0.43]		
TAS-20	0.22	0.11	0.19	2.08*	[0.01, 0.44]		
BPD Checklist total score	–0.02	0.05	–0.05	–0.41	[–0.12, 0.08]		
HCs group (n = 104)							
	B	*SE*	*Β*	*t*	95% CI for B	Adjusted R²	*F*
Constant	–6.73	4.27		− 1.58	–15.20	0.45***	17.84***
STAI-Trait	0.48	0.14	0.41	3.55***	0.21		
CESD-R	0.02	0.12	0.01	0.13	–0.22		
EDS	0.12	0.07	0.16	1.64	–0.03		
TAS-20	0.13	0.08	0.14	1.60	–0.03		
BPD Checklist total score	0.07	0.08	0.10	0.93	–0.08		

*Note.* STAI-Trait – trait anxiety measured by the State-Trait Anxiety Inventory; CESD-R – The Center for Epidemiologic Studies Depression Scale–Revised; EDS – Emotional Dysregulation Scale; TAS-20 – Toronto Alexithymia Scale; BPD Checklist – Borderline Personality Disorder Checklist

* *p* < 0.05. ** *p* < 0.01. *** *p* < 0.001

### Mediation analysis

Using data from the entire examined sample, we tested the multiple parallel mediation model to examine the relationship between BPD diagnosis and the level of self-disgust with alexithymia, emotion dysregulation, and comorbid psychopathology as mediators. The direct, indirect, and total effects of mediational bootstrapping analyses are reported in Table 4. A partial mediating effect of alexithymia, emotion dysregulation, trait anxiety, and depressive symptoms was found between BPD diagnosis and the level of self-disgust. The indirect effect accounted for 70% of the total effect.

**Table 4:** Direct, indirect, and total effects for parallel mediation models for the relationship between BPD diagnosis or BPD symptoms severity and the level of self-disgust

	B	*SE*	*t*	95% CI for B
Model 1 (BPD diagnosis → SDS)				
Total effect	32.37	1.53	21.15***	[29.36, 35.39]
Direct effect	9.56	2.42	3.94***	[4.77, 14.35]
Mediator: TAS-20	0.18	0.07	2.86**	[0.06, 0.31]
Mediator: EDS	0.16	0.06	2.75**	[0.05, 0.27]
Mediator: STAI-Trait	0.42	0.10	4.00***	[0.21, 0.62]
Mediator: CESD-R	0.13	0.06	2.12*	[0.01, 0.25]
Total indirect	22.82	2.32	9.84***	[18.16, 27.47]
via TAS-20	3.77	1.29	2.92**	[1.30, 6.44]
via EDS	5.22	2.18	2.47*	[0.94, 9.58]
via STAI-Trait	9.19	2.58	3.57***	[4.08, 14.13]
via CESD-R	4.63	2.39	1.94*	[0.08, 9.58]
Model 2 (BPD Checklist → SDS)				
Total effect	0.40	0.02	21.01***	[0.36, 0.43]
Direct effect	0.04	0.04	1.10	[–0.03, 0.11]
Mediator: TAS-20	0.22	0.07	3.23***	[0.09, 0.35]
Mediator: EDS	0.18	0.06	2.98**	[0.06, 0.30]
Mediator: STAI-Trait	0.44	0.11	3.92***	[0.22, 0.66]
Mediator: CESD-R	0.21	0.06	3.30***	[0.08, 0.34]
Total indirect	0.36	0.03	10.44***	[0.29, 0.42]
via TAS-20	0.06	0.02	3.41***	[0.02, 0.09]
via EDS	0.08	0.03	2.60**	[0.02, 0.14]
via STAI-Trait	0.13	0.03	3.74***	[0.06, 0.19]
via CESD-R	0.09	0.03	2.79**	[0.03, 0.16]

*Note.* CI = confidence interval; 5,000 bootstrap samples were used to generate bias-corrected 95% CI for the indirect effect; BPD diagnosis – diagnosis of borderline personality disorder (0 – HCs group; 1 – BPD group); SDS – self-disgust measured by Self-Disgust Scale; TAS-20 – alexithymia measured by Toronto Alexithymia Scale; EDS – emotion dysregulation measured by Emotion Dysregulation Scale; BPD Checklist – the severity of symptoms of BPD measured by BPD Checklist; STAI-Trait – trait anxiety measured by the State-Trait Anxiety Inventory; CESD-R – The Center for Epidemiologic Studies Depression Scale–Revised

*p < 0.05. **p < 0.01. ***p < 0.001

In the second parallel mediation model testing the association between the level of BPD symptoms and self-disgust with alexithymia, emotion dysregulation, and comorbid psychopathology as mediators, the level of BPD symptoms was not directly associated with self-disgust, but it was significantly indirectly related via mediators. The indirect effect accounted for 90% of the total effect.

## Discussion

### Self-disgust, emotional functioning, core and comorbid psychopathology in BPD inpatients vs. HCs

The current study allowed for a comprehensive examination of the role of self-disgust and its associations with alexithymia, emotion dysregulation, disgust sensitivity, and comorbid psychopathology in individuals with BPD. The first aim was to compare the level of self-disgust in a large sample of inpatients with BPD. In line with our hypothesis and with the results of other studies [9, 10, 22], self-disgust and all of its dimensions were increased in a clinical group as compared to HCs. Further, moderate to large associations between self-disgust and the severity of BPD symptoms were revealed, which is also consistent with the results of other research [23]. With regard to associations of self-disgust with specific BPD symptoms, the relationships with identity disturbance were particularly prominent in both examined groups. The association of identity disturbance with self-disgust corresponds with the results of Rüsch et al. [7], who observed more disgust-prone implicit self-concept in patients with BPD as compared to HCs. Our result is also in line with the studies on self-referential processing in BPD pointing to decreased self-esteem [66] and increased self-criticism [67]. Self-esteem and self-criticism are closely related to negative self-conscious emotions such as self-disgust [68]. It has also been observed that individuals with BPD may have the intention to avoid self-awareness due to a negative body-related shame-prone self-concept and increased self-focus attention [69]. Consistently, Biermann et al. [70] in their experimental study revealed that individuals with BPD experienced higher disgust when seeing one’s own picture as compared to HCs, which is in line with their negative self-image. Overall, these findings may support the importance of negative self-referenced emotions including self-disgust in BPD.

Consistent with the previous research, the current study found that emotion dysregulation and alexithymia were increased in patients with BPD. Medium and large associations were observed between self-disgust and emotion dysregulation in both BPD and HC groups, which is in line with Linehan’s understanding of the role of invalidating environments in the development of extreme emotional, behavioral, and cognitive dysregulation in BPD [6]. Within this theory, the foundations for dysfunctional behaviors observed in BPD are emotional vulnerability (i.e., heightened sensitivity and reactivity to emotional stimuli, and a slow return to a baseline level of emotional arousal) accompanied by avoidance of emotional cues and self-invalidation (i.e., adoption of characteristics from the invalidating environment) [4] that may include the component of self-directed disgust [71]. Emotion dysregulation comprises increased emotional vulnerability coupled with difficulties in effective emotion regulation. In this light, the link between self-disgust with emotion dysregulation, but also with the severity of BPD symptoms seems consistent with the knowledge on the clinical picture of this disorder. Additionally, the observed medium associations between self-disgust and alexithymia also align with the negative self-related processing in BPD. Because self-referenced processing requires the engagement of both cognitive and emotional components, deficits in appropriate identification and experience of emotions, including self-related emotions, could be associated with self-directed disgust. In addition, we found medium and large associations between self-disgust and the level of trait anxiety and depressive symptoms, which is in line with well-established knowledge on such associations in various samples [10, 60, 72]. Contrary to our hypothesis, no significant associations were found between general self-disgust and general disgust sensitivity in none of the examined groups.

Although it was not the main purpose of our study, it is worth mentioning the results on disgust sensitivity. Contrary to previous studies that have shown increased disgust sensitivity in individuals with BPD [7–9], our study found that general disgust sensitivity and disgust sensitivity in the animal-reminder domain were decreased in inpatients with BPD. However, it is important to note that the effect size was small. Although there is a lack of direct evidence indicating decreased disgust sensitivity in this group, some studies indirectly support our results. For example, it has been observed that patients with BPD may be less accurate at recognizing disgust from faces [73, 74]. This impaired facial disgust recognition, along with the previously discussed decreased disgust sensitivity, is consistent with the widely-supported research on increased alexithymia in BPD [75], as well as our own findings. Perhaps the general difficulty in identifying emotional cues in individuals with BPD may lead to a lower subjective assessment of the experienced disgust from external stimuli, as observed in our study. Another possible interpretation of the decreased disgust sensitivity in BPD can be explained within the framework of schema therapy [76]. According to this approach, one of the basic coping modes for patients with BPD is the so-called *Detached Protector*. This mode entails a state of psychological withdrawal characterized by avoidance of emotional experiences and disconnection from others [77]. From a cognitive-behavioral perspective, the reduced disgust sensitivity observed in our study (which was also confirmed in the animal-reminder domain related to stimuli associated with human mortality) [57] may be attributed to habituation resulting from repeated exposure to disgust-inducing stimuli in BPD (such as acts of self-mutilation or self-induced vomiting). Habituation may potentially result in reduced avoidance of negative stimuli in individuals with BPD. However, this hypothesis necessitates further investigation in future studies. It is important to note the limited direct evidence supporting reduced general disgust sensitivity in BPD from other research, as well as the small effect size of our findings. Therefore, caution should be exercised when interpreting our results regarding disgust sensitivity.

#### Mediational models of the associations between BPD and self-disgust

Further, our goal was to extend the extant knowledge on why individuals with BPD may have a higher level of self-disgust. To answer this question, we examined the mediational role of emotional dysfunctions and comorbid anxiety and depression in the association between BPD and self-disgust. In our first model alexithymia, emotion dysregulation, trait anxiety, and depressive symptoms partially mediated this association, accounting for 70% of the effect. Although this was not the full mediation, it can be interpreted that in BPD the level of self-disgust may be high mainly due to emotional alterations and comorbid anxiety and depressive symptoms, however, some part of the association could probably be explained by some other factors that were not examined within our study. Sansone et al. [78] observed that individuals with BPD tend to believe that attractiveness is an important factor for happiness and acceptance and that they show less comfort and trust in their own bodies. Considering the increasing evidence regarding the importance of body image disturbances in the psychopathology of BPD [79] and the fact that self-disgust seems to be associated with negative body image [80], we suggest that negative body image could explain another significant part of the association, however, this should be confirmed in future investigations.

Our second mediational model assessed the mediational effect of the constructs mentioned above on the relationship between BPD symptoms and self-disgust. In this case, alexithymia, emotion dysregulation, trait anxiety, and depression fully mediated this relationship and accounted for 90% of the effect. Consistent with the dimensional approach to personality disorder diagnosis, our results suggest that difficulties with emotion recognition, expression, and regulation, along with anxiety and depressive symptoms, largely explain the increased self-disgust in individuals with higher levels of BPD features. Carreiras et al. [32] found that self-disgust feelings had a significant effect on basal levels of BPD features and 12-month growth rates of these features in adolescents. Their findings suggest that self-disgust may influence the developmental trajectory of borderline features. However, our results provide some explanation for the causes of high levels of self-disgust in people with elevated BPD symptoms. Although our findings do not prove causal associations, they indirectly support the dimensional view of personality difficulties, as the process explained by our model may also occur in individuals who do not meet the categorical classification criteria. More pronounced BPD characteristics are associated with increased self-related disgust when emotional dysfunctions, depression, and anxiety are taken into account. These factors could contribute to the gradual exacerbation of psychological issues in these individuals, although this hypothesis would require confirmation in longitudinal studies.

### Limitations and future directions

Our study has several limitations. Firstly, we relied on self-report questionnaires in a cross-sectional design to investigate self-disgust and its associations with emotional functioning and psychopathological symptoms. However, BPD is characterized by frequent and intense affective, behavioral, interpersonal, and identity-related changes, and participants’ responses may not accurately reflect their daily-life functioning. Therefore, future studies should investigate momentary self-disgust in relation to daily changes in affect, emotion regulation strategies, and individual behavioral patterns. Furthermore, the cross-sectional design of our study does not allow for drawing conclusions regarding causality in the examined associations between variables. Secondly, we did not examine some of the disgust-related constructs that could be relevant to self-disgust, such as disgust propensity. Additionally, given the importance of disturbances in body image for the psychopathology of BPD, future studies focusing on self-disgust in this population should also examine this construct.

Although longitudinal investigations showing that self-disgust may be involved in the development of borderline features in adolescents have already been conducted [32], it would be worth examining how changes in self-disgust are related to the level of BPD symptoms in adults with BPD over time. Moreover, the inclusion of the recovered participants within such longitudinal investigations could help verify whether self-disgust remains a state- or trait-dependent clinical construct. Future research should also include clinical control groups. Considering that in our study, emotion dysregulation accounted for a significant proportion of variance in the relationship between BPD and self-disgust and that in previous studies self-disgust reached its highest levels in patient groups with BPD and AN [10], as well as considering the prevalence of contrasting maladaptive emotion regulation strategies in BPD (e.g., self-harm) and in the restrictive type of AN (e.g., food restriction), it would be worthwhile to investigate the role of maladaptive emotion regulation strategies in the development of self-disgust in these two disorders.

There are still few experimental studies investigating the role of negative self-conscious emotions in BPD. It would be especially valuable to examine the brain correlates of self-related disgust and the regulation of self-conscious emotions in BPD in functional magnetic resonance imaging. Finally, considering that shame may be involved in the development of self-disgust [81], self-directed disgust may serve as a mechanism through which shame is linked to psychopathological symptoms in other disorders [82], and both of these self-conscious emotions are associated with symptoms that might be present in the course of BPD (e.g., self-harm or identity disturbance) [68, 83, 84], it is worth investigating the role of both of these emotions simultaneously in relation to BPD.

## Conclusions

The present study is the first to examine the role of emotion dysregulation, alexithymia, and comorbid symptoms in the association between BPD and self-disgust. Women with BPD reported heightened levels of self-disgust, emotional dysfunction, and BPD-related core and comorbid characteristics, but decreased level of disgust sensitivity. Our results provide insight into the factors associated with increased self-disgust in individuals with heightened BPD symptoms. We found that emotion dysregulation, alexithymia, comorbid trait anxiety, and depressive symptoms partially mediate the relationship between BPD diagnosis and self-disgust, and fully mediate the association between the severity of BPD symptoms and self-disgust. In conclusion, our study suggests that difficulties in emotion processing and regulation, as well as comorbid psychopathology, may contribute to self-related disgust in BPD.

## Data Availability

The datasets used and analyzed during the current study are available from the corresponding author upon reasonable request.

## References

[1] American Psychiatric Association. Diagnostic and statistical manual of mental disorders: DSM-5. Vol. 5. American psychiatric association Washington, DC; 2013.

[2] Glenn CR, Klonsky ED (2009). Emotion dysregulation as a core feature of borderline personality disorder. J Personal Disord.

[3] Carpenter RW, Trull TJ (2013). Components of emotion dysregulation in borderline personality disorder: a review. Curr Psychiatry Rep.

[4] Linehan MM. Cognitive-behavioral treatment of borderline personality disorder. Guilford Publications; 1993.

[5] Carreiras D, Castilho P, Cunha M. What stands between self-disgust and borderline features? The need to cultivate self-compassion in adolescents from Portugal. Psychologica [Internet]. 2021 Dec 28 [cited 2022 Jun 28];64(2):51–64. Available from: https://impactum-journals.uc.pt/psychologica/article/view/10217.

[6] Crowell SE, Beauchaine TP, Linehan MM (2009). A biosocial developmental model of borderline personality: elaborating and extending linehan’s theory. Psychol Bull.

[7] Rüsch N, Schulz D, Valerius G, Steil R, Bohus M, Schmahl C (2011). Disgust and implicit self-concept in women with borderline personality disorder and posttraumatic stress disorder. Eur Arch Psychiatry Clin Neurosci.

[8] Schienle A, Schäfer A, Stark R, Walter B, Franz M, Vaitl D (2003). Disgust sensitivity in psychiatric disorders: a questionnaire study. J Nerv Ment Dis.

[9] Schienle A, Haas-Krammer A, Schöggl H, Kapfhammer HP, Ille R (2013). Altered state and trait disgust in borderline personality disorder. J Nerv Ment Dis.

[10] Ille R, Schöggl H, Kapfhammer HP, Arendasy M, Sommer M, Schienle A (2014). Self-disgust in mental disorders—symptom-related or disorder-specific?. Compr Psychiatry.

[11] Daros AR, Zakzanis KK, Ruocco A (2013). Facial emotion recognition in borderline personality disorder. Psychol Med.

[12] Unoka Z, Fogd D, Füzy M, Csukly G (2011). Misreading the facial signs: specific impairments and error patterns in recognition of facial emotions with negative valence in borderline personality disorder. Psychiatry Res.

[13] Kockler TD, Santangelo PS, Limberger MF, Bohus M, Ebner-Priemer UW (2020). Specific or transdiagnostic? The occurrence of emotions and their association with distress in the daily life of patients with borderline personality disorder compared to clinical and healthy controls. Psychiatry Res.

[14] Ebner-Priemer UW, Welch SS, Grossman P, Reisch T, Linehan MM, Bohus M (2007). Psychophysiological ambulatory assessment of affective dysregulation in borderline personality disorder. Psychiatry Res.

[15] Standish AJ, Benfield JA, Bernstein MJ, Tragesser S (2014). Characteristics of borderline personality disorder and disgust sensitivity. Psychol Rec.

[16] Rüsch N, Lieb K, Göttler I, Hermann C, Schramm E, Richter H (2007). Shame and implicit self-concept in women with borderline personality disorder. Am J Psychiatry.

[17] Skodol AE, Clark LA, Bender DS, Krueger RF, Morey LC, Verheul R (2011). Proposed changes in personality and personality disorder assessment and diagnosis for DSM-5 part I: description and rationale. Personal Disord: Theory Res Treat.

[18] Moncrieff-Boyd J, Byrne S, Nunn K (2014). Disgust and anorexia nervosa: confusion between self and non-self. Adv Eat Disord Theory Res Pract.

[19] Akram U, Stevenson JC (2021). Self-disgust and the dark triad traits: the role of expressive suppression. Personal Individ Differ.

[20] Powell PA, Simpson J, Overton PG (2015). An introduction to the revolting self: self-disgust as an emotion schema. The revolting self: perspectives on the psychological, social, and clinical implications of self-directed disgust.

[21] Clarke A, Simpson J, Varese F (2019). A systematic review of the clinical utility of the concept of self-disgust. Clin Psychol Psychother.

[22] Dudas RB, Mole TB, Morris LS, Denman C, Hill E, Szalma B et al. Amygdala and dlPFC abnormalities, with aberrant connectivity and habituation in response to emotional stimuli in females with BPD. J Affect Disord [Internet]. 2017 Jan 15 [cited 2022 Jun 28];208:460–6. Available from: https://www.sciencedirect.com/science/article/pii/S0165032716309454. 10.1016/j.jad.2016.10.043 27838143

[23] Schienle A, Leutgeb V, Wabnegger A (2015). Symptom severity and disgust-related traits in borderline personality disorder: the role of amygdala subdivisions. Psychiatry Res Neuroimaging.

[24] Klonsky ED, Muehlenkamp JJ (2007). Self-injury: a research review for the practitioner. J Clin Psychol.

[25] Smith NB, Steele AM, Weitzman ML, Trueba AF, Meuret AE (2015). Investigating the role of self-disgust in nonsuicidal self-injury. Arch Suicide Res.

[26] Schienle A, Schwab D, Höfler C, Freudenthaler HH (2020). Self-disgust and its relationship with lifetime suicidal ideation and behavior. Crisis.

[27] Wicker B, Keysers C, Plailly J, Royet JP, Gallese V, Rizzolatti G (2003). Both of us disgusted in my insula: the common neural basis of seeing and feeling disgust. Neuron.

[28] Craig AD (2009). How do you feel—now? The anterior insula and human awareness. Nat Rev Neurosci.

[29] Meaney R, Hasking P, Reupert A (2016). Borderline personality disorder symptoms in college students: the complex interplay between alexithymia, emotional dysregulation and rumination. PLoS ONE.

[30] Tragesser SL, Solhan M, Schwartz-Mette R, Trull TJ (2007). The role of affective instability and impulsivity in predicting future BPD features. J Pers Disord.

[31] Krawitz R (2012). Behavioural treatment of chronic, severe self-loathing in people with borderline personality disorder. Part 1: interrupting the self-loathing cycle. Australas Psychiatry.

[32] Carreiras D, Cunha M, Castilho P (2022). Trajectories of borderline features in adolescents: a three-wave longitudinal study testing the effect of gender and self-disgust over 12 months. Personal Individ Differ.

[33] Drea CA. Self-disgust in personality disorders: the role of childhood abuse and trauma, emotional invalidation and shame (Doctoral dissertation, UCL (University College London)). 2017.

[34] Nilsson M, Lundh LG, Westling S (2022). Childhood maltreatment and self-hatred as distinguishing characteristics of psychiatric patients with self‐harm: a comparison with clinical and healthy controls. Clin Psychol Psychother.

[35] Lazuras L, Ypsilanti A, Powell P, Overton P (2019). The roles of impulsivity, self-regulation, and emotion regulation in the experience of self-disgust. Motiv Emot.

[36] Mancke F, Herpertz SC, Kleindienst N, Bertsch K (2017). Emotion dysregulation and trait anger sequentially mediate the association between borderline personality disorder and aggression. J Pers Disord.

[37] Fitzpatrick S, Kuo JR (2016). The impact of stimulus arousal level on emotion regulation effectiveness in borderline personality disorder. Psychiatry Res.

[38] Taylor GJ, Bagby RM, Parker JD (1991). The alexithymia construct: a potential paradigm for psychosomatic medicine. Psychosomatics.

[39] Palser ER, Palmer CE, Galvez-Pol A, Hannah R, Fotopoulou A, Kilner JM (2018). Alexithymia mediates the relationship between interoceptive sensibility and anxiety. PLoS ONE.

[40] Deborde AS, Miljkovitch R, Roy C, Dugré-Le Bigre C, Pham-Scottez A, Speranza M (2012). Alexithymia as a mediator between attachment and the development of borderline personality disorder in adolescence. J Personal Disord.

[41] Preece DA, Mehta A, Becerra R, Chen W, Allan A, Robinson K (2022). Why is alexithymia a risk factor for affective disorder symptoms? The role of emotion regulation. J Affect Disord.

[42] Taylor GJ, Bagby RM (2013). Psychoanalysis and empirical research: the example of alexithymia. J Am Psychoanal Assoc.

[43] Powell PA, Simpson J, Overton PG (2013). When disgust leads to dysphoria: a three-wave longitudinal study assessing the temporal relationship between self-disgust and depressive symptoms. Cogn Emot.

[44] Gao S, Zhang L, Yao X, Lin J, Meng X (2022). Associations between self-disgust, depression, and anxiety: a three-level meta-analytic review. Acta Psychol (Amst).

[45] Fatimah H, Rappaport LM, Bornovalova MA (2023). Symptoms of borderline personality and related pathologies behave as temporal and contemporaneous networks. Pers Disord : Theory Res Treat.

[46] First MB, Benjamin LS, Spitzer RL, Williams JB. SCID-5-PD Ustrukturalizowany wywiad kliniczny do badania zaburzeń osobowości według DSM-5®: podręcznik klinicysty. American Psychiatric Association Publishing; 2018.

[47] Bloo J, Arntz A, Schouten E (2017). The borderline personality disorder checklist: psychometric evaluation and factorial structure in clinical and nonclinical samples. Ann Psychol.

[48] Grzegorzewski P. Validation of the Polish version of the Borderline Personality Disorder Checklist in a clinical and general sample [in preparation]. 2023.

[49] Spielberger CD, Gorsuch RL, Lushene R, Vagg PR, Jacobs GA (1983). State trait anxiety inventory for adults manual.

[50] Wrześniewski K, Sosnowski T, Matusik D (2002). Inwentarz Stanu i Cechy Lęku STAI. Polska adaptacja STAI. Podręcznik.

[51] Eaton WW, Smith C, Ybarra M, Muntaner C, Tien A. Center for Epidemiologic Studies Depression Scale: review and revision (CESD and CESD-R). 2004.

[52] Koziara K (2016). Assessment of depressiveness in population. Psychometric evaluation of the polish version of the CESD-R. Psychiatr Pol.

[53] Powers A, Stevens J, Fani N, Bradley B (2015). Construct validity of a short, self report instrument assessing emotional dysregulation. Psychiatry Res.

[54] Grzegorzewski P. Validation of the Polish version of the Emotion Dysregulation Scale in a clinical and general sample [in preparation]. 2023.

[55] Bagby RM, Parker JD, Taylor GJ (1994). The twenty-item Toronto Alexithymia Scale—I. item selection and cross-validation of the factor structure. J Psychosom Res.

[56] Ścigała DK, Zdankiewicz-Ścigała E, Bedyńska S, Kokoszka A (2020). Psychometric properties and configural invariance of the polish–language version of the 20-item toronto alexithymia scale in non-clinical and alcohol addict persons. Front Psychol.

[57] Haidt J, McCauley C, Rozin P. Individual differences in sensitivity to disgust: A scale sampling seven domains of disgust elicitors. Personal Individ Differ [Internet]. 1994 May 1 [cited 2022 Jun 29];16(5):701–13. Available from: https://www.sciencedirect.com/science/article/pii/0191886994902127.

[58] Olatunji BO, Williams NL, Tolin DF, Abramowitz JS, Sawchuk CN, Lohr JM (2007). The Disgust Scale: item analysis, factor structure, and suggestions for refinement. Psychol Assess.

[59] Kot E. Validation of the Polish version of the Disgust Scale– Revised in a general population sample [in preparation]. 2021.

[60] Overton P, Markland F, Taggart H, Bagshaw G, Simpson J (2008). Self-disgust mediates the relationship between dysfunctional cognitions and depressive symptomatology. Emotion.

[61] Kot E. Validation of the Polish version of the Self-Disgust Scale in a general population sample [in preparation]. 2021.

[62] George D, Mallery P. Frequencies. IBM SPSS Statistics 23 step by step. Routledge; 2016. 115–25.

[63] Field A. Discovering statistics using SPSS. 2009.

[64] Preacher KJ, Hayes AF. SPSS and SAS procedures for estimating indirect effects in simple mediation models. Behavior research methods, instruments, & computers. 2004;36:717 – 31. 10.3758/bf03206553 15641418

[65] Hayes AF. Mediation, moderation, and conditional process analysis. Introduction to mediation, moderation, and conditional process analysis: A regression-based approach. 2013;1:20.

[66] Bungert M, Liebke L, Thome J, Haeussler K, Bohus M, Lis S (2015). Rejection sensitivity and symptom severity in patients with borderline personality disorder: effects of childhood maltreatment and self-esteem. Borderline Personal Disord Emot Dysregulation.

[67] Kopala-Sibley DC, Zuroff DC, Russell JJ, Moskowitz D, Paris J (2012). Understanding heterogeneity in borderline personality disorder: differences in affective reactivity explained by the traits of dependency and self-criticism. J Abnorm Psychol.

[68] Winter D, Bohus M, Lis S (2017). Understanding negative self-evaluations in borderline personality disorder—A review of self-related cognitions, emotions, and motives. Curr Psychiatry Rep.

[69] Winter D, Koplin K, Lis S (2015). Can’t stand the look in the mirror? Self-awareness avoidance in borderline personality disorder. Borderline Personal Disord Emot Dysregulation.

[70] Biermann M, Schulze A, Vonderlin R, Bohus M, Lyssenko L, Lis S. Shame, self-disgust, and envy: an experimental study on negative emotional response in borderline personality disorder during the confrontation with the own face. Front Psychiatry. 2023;14. 10.3389/fpsyt.2023.1082785 PMC10030617 36970260

[71] McMain S, Korman LM, Dimeff L (2001). Dialectical behavior therapy and the treatment of emotion dysregulation. J Clin Psychol.

[72] Ypsilanti A, Lazuras L, Robson A, Akram U (2018). Anxiety and depression mediate the relationship between self-disgust and insomnia disorder. Sleep Health.

[73] Levine D, Marziali E, Hood J (1997). Emotion processing in borderline personality disorders. J Nerv Ment Dis.

[74] Vestergaard M, Kongerslev MT, Thomsen MS, Mathiesen BB, Harmer CJ, Simonsen E (2020). Women with borderline personality disorder show reduced identification of emotional facial expressions and a heightened negativity bias. J Personal Disord.

[75] New AS, Rot M, aan het, Ripoll LH, Perez-Rodriguez MM, Lazarus S, Zipursky E (2012). Empathy and alexithymia in borderline personality disorder: clinical and laboratory measures. J Personal Disord.

[76] Young JE, Klosko JS, Weishaar ME. Schema therapy: a practitioner’s guide. guilford press; 2006.

[77] Barazandeh H, Kissane DW, Saeedi N, Gordon M (2018). Schema modes and dissociation in borderline personality disorder/traits in adolescents or young adults. Psychiatry Res.

[78] Sansone RA, Chu JW, Wiederman MW (2010). Body image and borderline personality disorder among psychiatric inpatients. Compr Psychiatry.

[79] Wayda-Zalewska M, Kostecka B, Kucharska K (2021). Body image in borderline personality disorder: a systematic review of the emerging empirical literature. J Clin Med.

[80] von Spreckelsen P, Glashouwer KA, Bennik EC, Wessel I, de Jong PJ (2018). Negative body image: Relationships with heightened disgust propensity, disgust sensitivity, and self-directed disgust. PLoS ONE.

[81] Power M, Dalgleish T (1997). Cognition and emotions: from order to disorder.

[82] Olatunji BO, Cox R, Kim EH (2015). Self-disgust mediates the associations between shame and symptoms of bulimia and obsessive-compulsive disorder. J Soc Clin Psychol.

[83] Sheehy K, Noureen A, Khaliq A, Dhingra K, Husain N, Pontin EE (2019). An examination of the relationship between shame, guilt and self-harm: a systematic review and meta-analysis. Clin Psychol Rev.

[84] Unoka Z, Vizin G (2017). To see in a mirror dimly. The looking glass self is self-shaming in borderline personality disorder. Psychiatry Res.

